# The diagnostic value of midbrain hyperechogenicity in ALS is limited for discriminating key ALS differential diagnoses

**DOI:** 10.1186/s12883-015-0280-x

**Published:** 2015-03-14

**Authors:** Andreas Hermann, Ulrike Reuner, Jochen Schaefer, Panteha Fathinia, Tordis Leimert, Jan Kassubek, Mario Leimert, Albert C Ludolph, Alexander Storch

**Affiliations:** Division for Neurodegenerative Diseases, Department of Neurology, Technische Universität Dresden and German Center for NeurodegenerativeDiseases (DZNE), Research site Dresden, Fetscherstrasse 74, 01307 Dresden, Germany; Department of Neurology, Technische Universität Dresden, Dresden, Germany; Department of Neurology, University Ulm, Ulm, Germany; Department of Neurosurgery, Technische Universität Dresden, Dresden, Germany

**Keywords:** Motor neuron disease, Substantia nigra, Myasthenia gravis, Inflammatory neuropathies, Cervical canal stenosis

## Abstract

**Background:**

Hyperechogenicity of the substantia nigra was recently reported in patients with sporadic ALS with a frequency similar to PD. Data on the diagnostic utility compared to key differential diagnoses of ALS do not exist yet.

**Methods:**

We prospectively enrolled 43 patients with ALS, 29 with myasthenia gravis, 25 patients with inflammatory neuropathy, and 13 with cervical canal stenosis. All patients were examined by a blinded investigator using transcranial B-mode sonography planimetrically measuring hyperechogenic areas of the midbrain representing the substantia nigra.

**Results:**

Mean midbrain hyperechogenic area was increased in ALS compared to non-ALS differentials. ROC analysis revealed only small area under the curve for detecting ALS (AUC: 0.669 [95%CI: 0.56-0.78]; p = 0.006). Highest Youden index was observed for area size of <0.14 cm^2^ (Youden index: 0.28). Using this cut-off score and that generated from normative data of healthy controls, area size measurements provided a sensitivity of only 46-58% and specificity of 69-83% for detecting ALS. No correlations of hyperechogenic area sizes in ALS patients were found to age, gender, ALS subtype (bulbar versus spinal form), disease duration or ALS-FRS-R score.

**Conclusions:**

Midbrain hyperechogenicity is reproducibly found in ALS patients, but its diagnostic value for discriminating ALS from its key differentials is limited.

## Background

Transcranial B-mode sonography (TCS) of the midbrain is in the meantime well accepted for the differential diagnosis of Parkinson’s disease (PD) and various movement disorders [1-3]. Nine per cent of healthy 20–80 year old controls also show hyperechogenicity of the substantia nigra (SN) which is considered to be associated with a subclinical functional impairment of the nigrostriatal system [3]. We recently reported a similar high prevalence of hyperechogenecity of the substantia nigra in patients with amyotrophic lateral sclerosis (ALS) [4]. This was independently confirmed very recently [5]. No data exist yet on the diagnostic value in the differential diagnosis of motor neuron diseases (MND) compared to key clinical differentials of myasthenia gravis, inflammatory neuropathies and cervical canal stenosis.

Alteration of the regional iron metabolism is suspected to be the underlying neuropathological correlate of SN hyperechogenicity rather than the neurodegenerative process itself [6]. *Post-mortem* studies have reported a degeneration of the SN in sporadic ALS [7], which resembles stage 3 of the very recently reported neuropathological classification of Braak and Brettschneider [8,9]. Further preliminary studies reported increased iron content in ALS brain tissue [10]. Neuroimaging studies by the use of PET or SPECT have indeed shown abnormal pre-synaptic and post-synaptic striatal dopaminergic function in ALS patients [11,12].

We here prospectively recruited major differential diagnoses of MNDs to evaluate the diagnostic value of SN hyperechogenicity measured by transcranial sonography.

## Methods

### Subjects

Patients were recruited at the outpatient clinic of the Department of Neurology of the University Hospital Dresden from 2011 to 2013. These included subjects with definite or probable ALS according to the revised El Escorial criteria, antibody proven myasthenia gravis (MG), inflammatory neuropathies (IN) (including Guillain-Barré syndrome, chronic inflammatory demyelinating polyneuropathy, multifocal motor neuropathy) (clinical diagnosis according to European Federation of Neurological Societies and the Peripheral Nerve Society (EFNS/PNS) [13,14]) and cervical canal stenosis (CCS) (clinical diagnosis according to [15]). The study was approved by the institutional review board (EK 11012012; EK 182062012). Altogether, we included the following 110 subjects: 43 with ALS, 29 with myasthenia gravis, 25 with inflammatory neuropathy and 13 with cervical canal stenosis. Demographic and clinical characteristics of the study cohorts are displayed in Table 1.

**Table 1:** **Demographic and clinical data of ALS patients and differentials**

	**ALS**	**MG**	**IN**	**CCS**	***P*** **value**
No. of patients, n	43	29	25	13	
Female, n (%)	21 (49%)	12 (41%)	9 (36%)	6 (46%)	0.779*
Age (yr), Mean ± SD (range)	64.9 ± 9.6 (42–78)	61.7 ± 14.5 (34–83)	60.6 ± 16.1 (22–80)	62.5 ± 10.7 (48–81)	0.554^#^
ALS subtype					
Bulbar, n (%)	11/43 (17%)	-	-	-	-
Spinal, n (%)	28/43 (65%)	-	-	-	-
ALS-FRSR, mean ± SD (range)	33.6 ± 9.45 (14–48)	-	-	-	-
MG subtype					
Ocular, n (%)	-	6/28 (21%)	-	-	-
Bulbar, n (%)	-	4/28 (14%)	-	-	-
Generalized, n (%)	-	17/28 (61%)	-	-	-
IN subtypes					
CIDP	-	-	13/25 (52%)	-	-
GBS	-	-	4/25 (16%)	-	-
MMN	-	-	4/25 (16%)	-	-
MADSAM	-	-	2/25 (8%)	-	-
AMSAN	-	-	1/25 (4%)	-	-
Mononeuritis muliplex	-	-	1/25 (4%)	-	-
CCS					
Degree; absolute/high	-	-	-	7/3	-
Myelopathy, n (%)	-	-	-	10/12 (83%)	-
Paraspasticity, n(%)	-	-	-	3/11 (27%)	-

*Fisher exact test; ^#^One-way two-sided ANOVA. Denominators may differ due to missing data in some individuals.

ALS, amyotrophic lateral sclerosis; MG, Myasthenia gravis; IN, inflammatory neuropathies; CIDP, chronic inflammatory demyelinating polyneuropathy; GBS, Guillain-Barré-Syndrome; MMN, multifocal motor neuropathy; MADSAM, multifocal acquired demyelinating sensory and motor neuropathy; AMSAM, acute motor and sensory axonal neuropathy; CCS, cervical canal stenosis.

### Transcranial brain sonography

Data acquisition was performed by a blinded rater. Transcranial B-mode sonography (TCS) was performed by the use of a Toshiba Aplio MX, SSA-780A, with a 3 MHz probe located on the temporal bone window. The technical settings were depth 16 mm, brightness variable, dynamic range 45 dB. Each side was measured in three different planes, one above and one below the orbitomeatal line, parallel to the orbitomeatal line and the mean value was used for further analysis. The ipsilateral hyperechogenic SN was measured planimetrically in the midbrain according to standardized techniques as described by Berg and colleagues [3].

### Statistical analyses

Data were prospectively analyzed using the software program SPSS version 19.0 (SPSS Inc., Chicago, IL). Data are displayed as mean ± standard deviation (SD) or numbers (%), significance level was set at *P* < 0.05 (two-sided). ROC plots including calculation of the area under the curve (AUC) were used to display sensitivity and specificity of TCS measures for detection of ALS. For generation of cut-off values from ROC analysis, we used the Youden index as a measure of the theoretical optimum of test scores. Normal values of SN echogenic size were obtained in healthy controls by calculating the mean + 2 × SD leading to a normal range of <0.20 cm^2^ as reported previously [4] and perfectly fitting to the literature [3]. For the classification of patients with respect to their SN echogenicity, the mean value of bilateral measurements was used. Statistical comparisons of variables were calculated using *χ*^2^ test or one-way ANOVA with Bonferroni *post-hoc t-*test (see Results sections for details). Spearman’s rank correlation coefficient ρ was used to examine correlations (ρ < 0.3 was considered a weak, ρ = 0.3-0.59 a moderate, ρ ≥ 0.6 a strong correlation).

## Results

**Figure 1:**
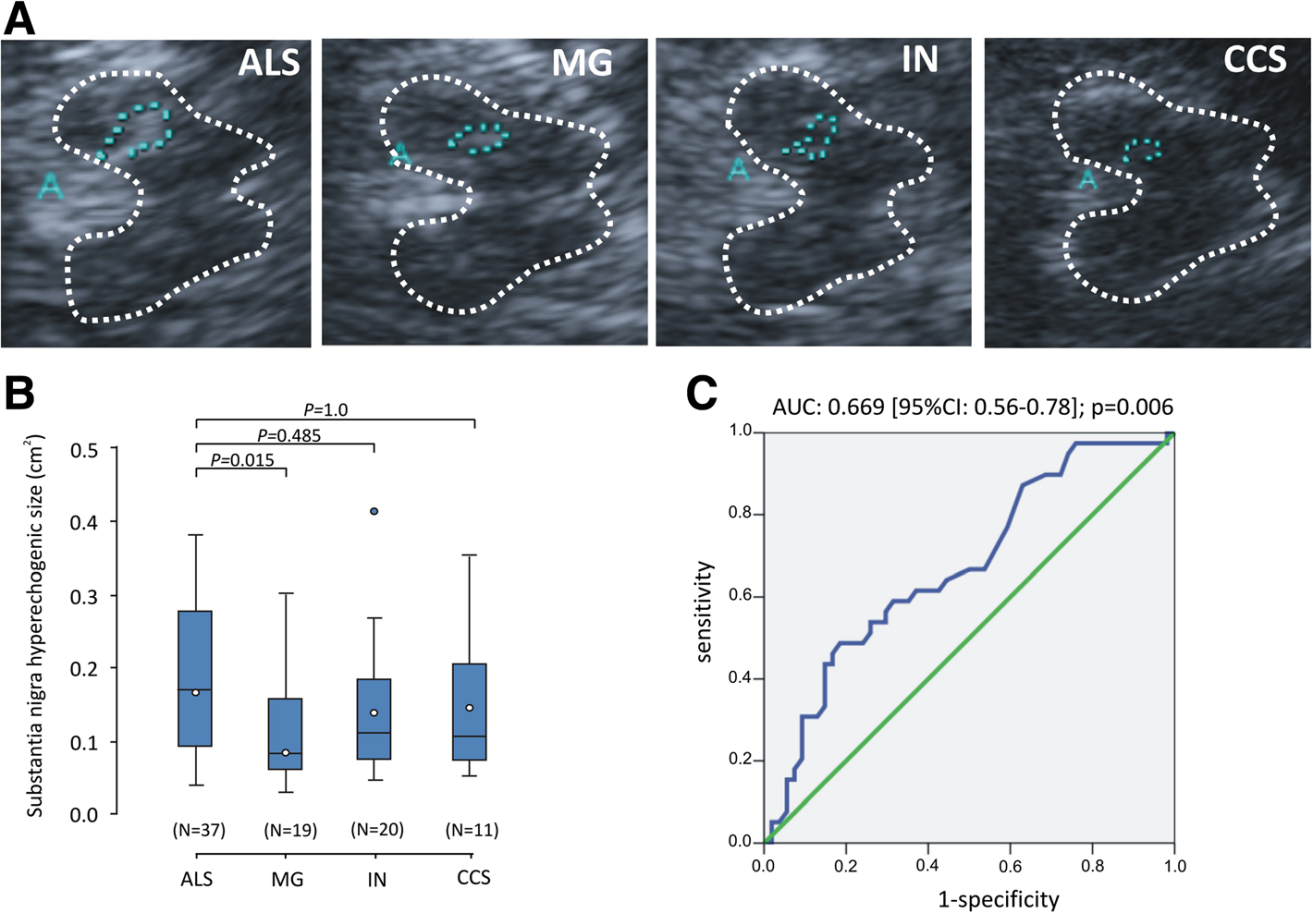
**Transcranial sonography (TCS) studies in ALS compared to myasthenia gravis (MG), inflammatory neuropathies (IN) and cervical canal stenosis (CCS). (A)** Representative TCS pictures of axial transsections of the brain at midbrain level in one patient with ALS, MG, IN and CCS, respectively. The TCS images show abnormal SN in ALS. In the area of the substantia nigra (SN), a marked hyperechogenicity can be seen. **(B)** Box plots of SN areas measured by TCS in ALS, MG, IN and CCS patients. The plots show the 10th percentile, first quartile, median, third quartile, and 90th percentile for each parameter. Open circles represent the means. Numbers in parentheses indicate the numbers of patients analyzed. *P* values are from Bonferroni adjusted *post-hoc t*-tests. **(C)** Receiver operating characteristics (ROC) curves displaying the sensitivity and specificity of SN hyperechogenic area for diagnosis of ALS. Insets indicate AUC values, 95% confidence intervals and statistics.

A temporal bone window sufficient for an adequate sonographic analysis of the SN at least on one side was found in 37 of 43 (86%) ALS patients, 20 of 29 (69%) MG patients, 22 of 25 (88%) IN patients, and 12 of 13 (92%) CCS patients (*P* = 0.139; *χ*^2^ test). In subjects with sufficient temporal acoustic bone windows, mean midbrain hyperechogenic areas were significantly higher in ALS compared non ALS patients (*P* = 0.006; unpaired two-sided *t*-test:). Compared to the various differential diagnoses, ALS patients had only significant higher mean SN area compared to MG patients (*P* = 0.015, Bonferroni adjusted post-hoc *t*-test), but not to all other patients with no significant differences between the differential diagnoses (one-way ANOVA: F-value = 3.4; *P* = 0.021; Figure 1B).

ROC analysis revealing sensitivities and specificities of any particular cut-off value of hyperechogenic SN area size to detect ALS is displayed in Figure 1C. The intervals between cut-off values for area size were between 0.27 cm^2^ (90th percentile specificity) and 0.07 cm^2^ (90th percentile sensitivity). The highest Youden index was observed at <0.14 cm^2^ (Youden index: 0.28). Using the cut-off from normative data from healthy controls of <0.20 cm^2^ [4], hyperechogenicity was found in 46% (17/37) of ALS patients, in 5% (1/20) of MG patients, in 23% (5/22) of IN patients and in 25% (3/12) of CCS patients, representing a sensitivity of 46% (95%CI: 30-63%) and specificity of 83% (95%CI: 70-91%) to detect ALS in our MND mimics cohort. The sensitivity and specificity values obtained at the maximal Youden index were similar with 58% sensitivity (95%CI: 41-73%) and 69% specificity (95%CI: 55-81%), respectively.

Neither age, gender, MND subtype, ALSFRS-R score, disease duration (r-0.136, p = 0.45) nor pathologically elevated central motor conduction time in magnetic evoked potentials correlated with hyperechogenic SN area.

## Discussion

We report data on the diagnostic value of regional midbrain hyperechogenicity determined by TCS in ALS compared to the key differential diagnoses myasthenia gravis, inflammatory neuropathies, and cervical canal stenosis. We showed increased echogenicity of the SN in ALS compared to non ALS differentials, but its diagnostic value is limited, questioning this simple and non-invasive technique as a useful additional technical tool in the differential diagnosis of motor neuron diseases. Hyperechogenic area size in ALS patients did not correlate with age, gender, ALS-FRSR score or disease subtype.

The diagnosis of MND is still done by exclusion of other diseases which have to be considered, as these are for example myasthenia gravis, inflammatory neuropathies and cervical canal stenosis. In some cases this can be particularly difficult in early stages of the disease. Therefore, any diagnostic finding being able to distinguish these challenging differential diagnoses are helpful for the early diagnosis of motor neuron disease. Showing a specificity of >80% using the standard cut-off value TCS seem to be helpful for this purpose, however the sensitivity is rather low (46%). Major limitations of our study are the mono-centric recruitment of patients at a specialized MND centre with a more strongly represented MND patient cohort compared to the mimics cohorts, the rather limited sample size of cervical canal stenosis as an important differential diagnosis the lacking intra-/inter-rater variability measurements.

Our sonography data fits to early pathological studies showing neurodegeneration of the SN region [7] and increased iron content in sporadic ALS brain tissue [10]. Disease spreading to non-motor systems including the midbrain regions was recently reported underlining the hypothesis of a neuronal multisystem disorder [8,9]. Also other neuroimaging techniques revealed functional impairment of the dopaminergic nigro-striatal pathway [11,12]. Recently, hypointense signals in the motor cortex were reported in routine cMRI [16]. In a pilot study using 7 T MRI, this hypointense signal could be localized to deeper cortical layers and – in very preliminary post-mortem analysis – iron depositions (mainly in microglial cells) was found in deeper cortical layers [17]. We do not yet know whether these findings are due to similar neuropathology events as in PD in which the SN hyperechogenicity is due to increased iron content of the midbrain [6].

## Conclusion

In summary, substantia nigra hyperechogenecity is reliably and reproducibly observed in ALS patients, but its diagnostic value in discriminating ALS from key ALS mimics is limited. Nevertheless, its value in the diagnostic process of ALS could become one additional cobblestone if one is aware of its limitations.

### Ethic committee approval

The study was approved by the local ethics committee of the University of Dresden.
